# Successful Fecal Microbiota Transplants in Post-antibiotic Treated Recurrent *Clostridioides difficile* Patients Induce Acylcarnitine and Sphingolipid Lipidomic Shifts

**DOI:** 10.21203/rs.3.rs-7888346/v1

**Published:** 2025-11-03

**Authors:** Abigail S Gancz, Guozhi Zhang, Arthur S McMillan, Michael K Dougherty, Sarah K McGill, Ajay S Gulati, Erin S Baker, Casey M Theriot

**Affiliations:** 1 Department of Population Health and Pathobiology, College of Veterinary Medicine, North Carolina State University, Raleigh, North Carolina, USA; 2 Department of Chemistry, University of North Carolina at Chapel Hill, Chapel Hill, North Carolina, USA; 3 Department of Biology, University of North Carolina at Chapel Hill, Chapel Hill, North Carolina, USA; 4 Department of Medicine, Division of Gastroenterology and Hepatology, University of North Carolina at Chapel Hill, Chapel Hill, North Carolina, USA; 5 Rex Digestive Healthcare, Raleigh, North Carolina, USA; 6 Department of Pediatrics, Division of Gastroenterology and Hepatology, University of North Carolina at Chapel Hill, Chapel Hill, North Carolina, USA; 7 Department of Pathology and Laboratory Medicine, University of North Carolina at Chapel Hill, Chapel Hill, North Carolina, USA

## Abstract

*Clostridioides difficile* infection (CDI) is an urgent public health threat in the United States, resulting on an annual basis in over half a million cases, more than 29,000 deaths, and $4.8 billion in healthcare costs. While fecal microbiota transplants (FMTs) have proven more effective than standard-of-care antibiotics in resolving recurrent CDI (rCDI), their inherent risks underscore the need for advancements in regulated alternative therapies such as live biotherapeutic products (LBPs). The development of effective LBPs, however, is contingent upon better understanding the biological mechanisms underlying FMT efficacy. Building on our previously published untargeted metabolomic study which identified lipids as major explanatory factors associated with successful FMTs, we assessed additional lipid species using an instrumental platform coupling liquid chromatography, ion mobility spectrometry, collision induced dissociation, and mass spectrometry (LC-IMS-CID-MS) techniques. This platform and data analysis workflow enable the evaluation of >850 unique lipid species across 26 classes. Here, we confidently identified 397 lipids in the stools of 15 rCDI patients at pre- and post-FMT (2 week, 2 month, and 6 month) time points. Statistical evaluations of the lipidomic data illustrated that FMT-administration drastically reshapes the lipidome (adonis test, R^2^=0.11999, Pr(>F) <0.001), including 96 specific lipid species across 18 lipid classes (mixed effects modeling, BH correction, p < 0.05). In particular, we noted that medium and long-chain acylcarnitines decreased following FMT administration, while very long-chain acylcarnitines were elevated in post-FMT samples. Additionally, we observed assayed sphingolipids to be elevated pre-FMT with the exception of trihydroxy ceramides, which were highly upregulated post-FMT. These lipidomic alterations suggest that FMT administration may influence intestinal barrier integrity, inflammatory signaling, or apoptosis pathways. Interestingly, there was a strong co-occurrence of medium and long-chain acylcarnitines with Enterobacteriaceae, a bacterial family that has been demonstrated to utilize carnitine for growth. These findings highlight the critical role of the lipidome in patient susceptibility to rCDI and suggest the interactions between microbiota and lipids pre- and post-FMT as targets for developing next-generation LBPs.

## Introduction

*Clostridioides difficile* infection (CDI) is a leading cause of diarrheal diseases globally and a major public health threat, with rising mortality rates, morbidity burdens, and economic tolls^1,2^. In the United States alone, CDI is associated with nearly half a million annual infections, >29,300 deaths, and $4.8 billion in healthcare costs^3,4^. While CDI is commonly known for its prevalence in hospital settings, community-acquired infections have steadily increased to account for a large proportion of cases^5^. Clinical manifestations of CDI vary from asymptomatic colonization to diarrhea to more severe and debilitating conditions with risks of sepsis, toxic megacolon, and renal failure^6^. One of the most challenging aspects of managing CDI is that, even after receiving standard-of-care primary treatments (typically the antibiotics vancomycin or fidaxomicin), an average of 30% of patients will experience increasingly severe episodes of recurrent CDI (rCDI)^7–10^. These high rCDI rates are associated with antibiotic-induced disruptions of the gut microbiota, which erode microbiota-derived mechanisms of colonization resistance (i.e., the ability of the gut microbiota to prevent the colonization and growth of the pathogen)^11,12^.

The current standard-of-care treatment for restoring colonization resistance in rCDI patients are microbiota-focused therapeutics (MFTs), defined as refined fecal microbiota transplants (FMTs)^13^. Unlike traditional FMTs, which are highly effective^14–16^ but limited by safety, availability, and standardization challenges, MFTs are considered effective, safe, and accessible treatments for most patients, excluding fulminant and pediatric patients^17,18^. However, both FMTs and MFTs serve as broad-spectrum interventions with only partially understood mechanisms of action, limiting their ability to be optimized and personalized^19^. Therefore, a growing body of clinicians and researchers are moving toward the development of non-stool derived, live biotherapeutic product (LBP) therapies^20,21^ which would comprise of designed microbes or microbial consortia with known mechanisms of restoring colonization resistance. While a limited number of such products are currently being trialed^22,23^, a significant barrier to advancing these next generation therapeutics is the incomplete understanding of the microbiota and metabolite-derived colonization resistance mechanisms driving FMT and MFT success.

Although many mechanisms of colonization susceptibility and resistance remain unknown in CDI, metabolomics has recently been utilized to further understand resulting molecular changes. By profiling small molecule metabolites within cells, tissues, or organisms, metabolomics provides insights into physiological and biochemical processes. Untargeted metabolomic studies have been further useful as they aim to profile a wide array of metabolites without prior bias. However, untargeted metabolomic studies do have limitations with respect to targeted analyses. Targeted studies focus on predefined classes of metabolites and often have enhanced sensitivity and specificity over untargeted studies. While this allows more precise identification and quantification, any metabolite not targeted also cannot be assessed in that study. With respect to FMTs, researchers have historically employed targeted analyses using a variety of analytical techniques including gas chromatography-mass spectrometry (GC-MS) and liquid chromatography-mass spectrometry (LC-MS) to assess a variety of metabolites including bile acids^13,24–26^, short-chain fatty acids (SCFAs)^24^, carbohydrates, and amino acids. Among these, bile acid metabolomics has been particularly informative. Specifically, a multitude of studies have repeatedly demonstrated an FMT induced transition from primary to secondary bile acids^27–32^, and these observations have shed light on the importance of diverse microbial functions such as bile salt hydrolases (BSHs), bile acid inducible (*bai*) operons, and microbe host signaling to the restoration of colonization resistance. Targeted SCFA metabolomics have also shown post-FMT increases in these metabolites, which are also known to influence *C. difficile in vitro*^24,31^.

Since the scope of FMT associated metabolomic studies is continuously expanding, especially as new host and microbial-derived metabolites are described and discovered, the need for untargeted analyses continues to increase. For example, the discovery of a previously unknown spectrum of amino acid conjugations of host bile acids has expanded the set of detectable bile acid metabolites from dozens to hundreds^33,34^. Moreover, many of these microbially conjugated bile acids (MCBAs) have since been demonstrated to have significant impacts on the *C. difficile* life cycle^35^. While most FMT associated metabolomic studies have focused on targeted bile acid metabolomics, it has become increasingly evident that another important yet critically understudied class of metabolites that may offer mechanistic insights into FMT efficacy is lipids.

Lipids not only underlie many known mechanisms of colonization susceptibility and resistance (e.g., bile acid metabolism) but are likely to be critical components of others. As fundamental components of microbial and host cell membranes, lipids also serve as energy storage, signaling molecules, and nutrient absorbers. Lipid regulated physiological processes include inflammatory signaling cascades, such as those involving Toll-like receptors and G-protein-coupled receptors, as well as immunological modulations and intercellular communications between host and microbiota^36–38^. The metabolic diversity of lipids makes them dynamic substrates from both host- and microbe-derived enzymatic activity, thereby both reflecting and shaping intestinal ecologies. Preliminary evidence has increasingly indicated the importance of lipidomic shifts in colonization resilience restoration. For instance, research has demonstrated significant shifts in various lipid classes during CDI and rCDI^39,40^. Additionally, untargeted metabolic profiling conducted by our group revealed that lipids are some of the most substantially altered metabolites following FMT, indicating profound remodeling of gut metabolic networks and host-microbe interactions^31^. Specifically, our team identified acylcarnitine lipids to be major differentiators between pre- and post-FMT samples, with acylcarnitines decreasing after treatment. However, despite these insights, the untargeted metabolomic approach did not capture as wide or as detailed an array of lipids as other methods are capable of resolving. Therefore, in order to expand our knowledge of lipidomic changes, we utilized an instrumental platform combining liquid chromatography, ion mobility spectrometry, collision induced dissociation and mass spectrometry (LC-IMS-CID-MS) capable of assessing ~850 lipid species across 26 lipid classes. Unlike previously described methods including conventional LC-MS approaches, our method integrates high-resolution ion mobility separation with collision cross-section (CCS) matching, allowing for the resolution of isobaric and isomeric lipids. Additionally, our workflow also incorporates a custom spectral library with LC retention times, IMS collision cross sections, CID fragmentation patterns and MS precursor masses. This 4-dimensional database enables confident structural annotation of lipids that are otherwise unresolved by mass or retention time alone. Thus, this technique represents a substantial improvement in resolution and breadth in comparison to our previous lipidomic investigations.

Here, we applied the LC-IMS-CID-MS platform to identify and longitudinally track lipid profiles in fecal samples from 15 antibiotic-treated rCDI patients undergoing FMT^31^. We hypothesized that lipidomic shifts post-FMT would be indicative of functional changes in the gut mechanistically associated with colonization resistance. To explore this, we integrated the new lipidomic dataset with existing metagenomic data. Here, we show that FMT induces marked changes in lipid composition, and that medium- and long-chain acylcarnitines, together with trihydroxy ceramides, are among the strongest drivers of these shifts. We also found that Enterobacteriaceae, particularly *Klebsiella*, tracked closely with medium- and long-chain acylcarnitines, with Lachnospiraceae exhibiting inverse trends. Functionally, *Klebsiella* and *Escherichia* dominated amino acid biosynthesis and utilization pre-FMT, while *Blautia* and other Lachnospiraceae were associated with post-FMT biosynthetic potential. Together, these results exhibit novel FMT associated lipidomic trends, while highlighting critical mechanistic gaps prerequisite for the design of next generation therapeutics.

## Materials & Methods

### Metagenomic Analysis

**Figure 1:**
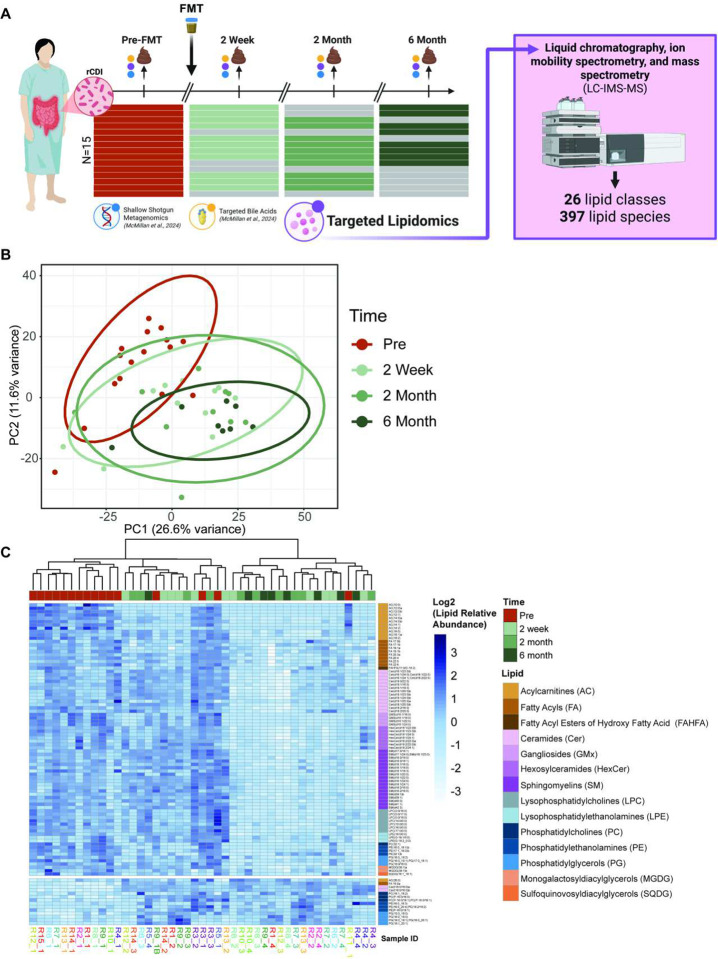
FMT significantly alters the lipidome of post-antibiotic rCDI patients. (A) Schematic of FMT stool sample collection from patients (n=15) undergoing FMT for rCDI. Fecal samples were collected pre-FMT (n=16 *due to one failure and re-administration), at 2 weeks (n=11), at 2 months (n=10), and at 6 months (n=8) for a total of (n=46) samples. Shallow shotgun metagenomics and targeted bile acid data were previously reported (McMillan et al., 2024), and for this study, targeted lipidomic coupled liquid chromatography, ion mobility spectrometry, and mass spectrometry (acronym) were used to isolate 397 lipid species from 26 classes. (B) Principal component analysis (PCA) of log2 transformed, 1e6 scaled, minimum-imputed lipids colored by timepoint of sample collection. (C) Heatmap of associations between sample collection time and the lipids that significantly associate with this variable, as determined by mixed effect modeling. Data were Z-score normalized by lipid species, and lipid species were grouped into clusters based on dendrogram branch cutting (k=2) and then ordered by lipid class. (Figure 1A was created in BioRender. Gancz, A. (2026) https://BioRender.com/kpxo64u)

Data previously generated by McMillan et al., 2024 for 45 human fecal samples (Table S1, Figure 1A) was used for this study. The raw sequencing reads were obtained from the published repository (SRA; BioProject PRJNA1055134). KneadData (v0.12.0) was used to remove reads mapping to the human genome, and the remaining reads were profiled for relative bacterial abundance using the biobakery pipeline, making use of MetaPhlAn^41^ (v4.1.1) and HUMAnN^42^ (v3.9), in combination with the CHOCOPhlAnSGB (mpa_vJun23_20403) database. All work involving these data was reviewed and approved by the UNC Institutional Review Board (protocol #16–2283).

### Glycerophospholipid, Sphingolipid, Glycerolipids, and Fatty Acid Lipid Extraction from Fecal Samples

#### Sample Preparation and Homogenization

Each fecal sample was weighed into 2 mL Omni microtubes containing 2.38 mm metal beads (Omni International, Kennesaw, GA, USA; catalog no. 19–620) with the exact sample weight recorded for data normalization as provided in Table S2. A 1 μL aliquot of SplashMix internal standard (Avanti Polar Lipids, Alabaster, AL, catalog no. 330707) was added to each tube to monitor extraction efficiency and reproducibility. Samples were homogenized in 350 μL LC-MS grade methanol (Fisher Scientific) using a Fisherbrand^™^ Bead Mill 24 Homogenizer (Fisher Scientific, Hampton, NH, USA; catalog no. 15-340-163) for a 30 s cycle at a speed of 4.2 m/s at room temperature. To prevent heat-induced lipid degradation, samples were cooled on ice between homogenization cycles.

#### Lipid Extraction and Phase Separation

Following homogenization, 700 μL LC-MS grade chloroform (Fisher Scientific) was added. Samples were vortexed at 2,000 rpm for 1 minute at room temperature, then sonicated on ice for 15 minutes to facilitate lipid extraction. Samples were centrifuged at 12,000 × g for 10 min at 4°C, to separate the insoluble feces parts and 800 μL of the supernatant was transferred to a SafeSeal microcentrifuge tubes (1.7 mL, Sorenson BioScience, Inc., Salt Lake City, UT, USA; catalog no. 39640T) for liquid-liquid phase extraction. Phase separation was achieved by adding 160 μL LC-MS grade ultrapure water (Fisher Scientific), followed by vortexing at 2,000 rpm for 1 minute at room temperature, sonication on ice for 15 minutes, and centrifugation at 12,000 × g for 5 minutes at 4°C. A 400 μL aliquot of the bottom lipid-containing organic phase was then collected.

To ensure complete lipid recovery, a second extraction step was performed by adding 600 μL chloroform to the remaining aqueous phase, followed by sonication for 15 minutes on ice and centrifugation at 12,000 × g for 5 minutes at 4°C. The additional 600 μL aliquot of the lower organic phase was collected and combined with the previous lipid extract for a total of 1,000 μL. The combined lipid extracts were dried under vacuum using a Savant SpeedVac SPD 13DLX vacuum concentrator (Thermo Scientific, Waltham, MA, USA) before LC-IMS-CID-MS analysis. The dried lipids were then resuspended in 30 μL chloroform and 80 μL methanol immediately to prevent oxidation.

#### Blanks

To assess extraction efficiency and reproducibility, we processed blank samples in parallel with FMT samples. Three extraction blanks (one with 50 μL of ultrapure water, one with 50 μL of phosphate-buffered saline (PBS), and one empty microtube) taken through all the steps above to assess potential contamination and ensure extraction consistency.

#### LC-IMS-CID-MS Analysis of Lipid Extracts

Extracted lipid samples were analyzed with a LC-IMS-CID-MS platform consisting of an Agilent 1290 Infinity UPLC system coupled to an Agilent 6560 IM-QTOF mass spectrometer (Agilent Technologies, Santa Clara, CA)^43–45^. Prior to analysis, mass calibration and CCS calibration were performed using the Agilent ESI-L Low Tuning Mix (Santa Clara, CA; catalog number G1969–85000). This ensured accurate *m/z* calibration and precise CCS measurements. Additionally, Brain Total Lipid Extract (BTLE) (Avanti Polar Lipids, Alabaster, AL, USA; catalog no. 131101) was used to benchmark instrumental performance. For analysis, the BTLE stock was diluted to 1 mg/mL in a 30:80 (v/v) chloroform/methanol mixture, and 10 μL of the diluted sample was injected onto the LC column. The three blank samples (described above) were also analyzed to evaluate any contamination from the extraction.

#### Chromatographic Separation

For RPLC analyses using the using an Agilent 1290 Infinity UPLC system, the lipids were separated on a Waters Acquity UPLC CSH C18 column (1.7 μm, 3.0 × 150 mm; part number 186005302, Waters, Milford, MA) maintained at 30°C. Mobile phase A (MPA) consisted of a 40:60 (v/v) mixture of LC-MS grade acetonitrile and water containing 10 mM ammonium acetate, while mobile phase B (MPB) was composed of a 90:10 (v/v) mixture of LC-MS grade isopropanol and acetonitrile with 10 mM ammonium acetate. Each sample was injected at a volume of 10 μL, and chromatographic separation was performed at a constant flow rate of 0.25 mL/min. The full LC gradient, including time points and solvent composition, is detailed in Table S3.

#### Ion Mobility Spectrometry and Mass Spectrometry Settings

To enhance additional identification confidence and interference removal, IMS was incorporated into the LC-CID-MS workflow. Lipid ions were separated based on their gas-phase mobility using a 78 cm drift tube with nitrogen as the buffer gas maintained at 3.95 Torr. Data acquisition was performed across a mass range of 50–1700 *m/z* in both positive and negative ionization modes, with additional MS parameters provided in Table S4. Drift time measurements were calibrated using ESI tuning mix to derive CCS values, ensuring accurate structural characterization. Moreover, the ion funnel trap fill time was set to 10 ms in the analyses, and a collision-induced dissociation (CID) ramp was dynamically adjusted based on drift times^43,46^ (Table S5).

### Data Processing and Analysis

#### Lipid Normalization and Pre-Processing

Raw IMS-MS data files (.d files) were drift time calibrated to CCS values using the Agilent ESI tuning mix and processed with the MassHunter IM-MS Browser software (version 10.0, Agilent Technologies). The processed data were subsequently imported into Skyline software^47^ (version 23.1.8, MacCoss Lab, Seattle, WA; release date 09/24/2023) for targeted lipid peak detection using a validated lipid spectral library. Here, 174 lipid species were detected in all samples in positive ion mode and 278 in negative mode. Peak areas are reported as area-under-the-curve (AUC). Moreover, lipid identifications exhibiting mass errors exceeding ±10 ppm were excluded from further analysis. A comprehensive list of identified lipid classes, including their abbreviated names and ionization modes used for analysis, is provided in Table 1. To account for variability in sample weight, peak areas were adjusted based on initial fecal mass. For comparative analysis, data normalization was performed using total ion chromatograph (TIC) values, and the resulting data are available in Table S6. Lipid values were transformed into log_2_, scaled (1 × 10^6^), and underwent minimum data imputation using R^48^ (v4.3.2) via RStudio^49^ (v2024.12.0).

#### Taxonomic and Functional Inference Pre-Processing

Taxonomic profiles generated as described above were imported into R^48^ (v4.3.2) via RStudio^49^ (v2024.12.0). Species-level subsets were created for specific bacterial families of interest. Metabolic pathway profiles were generated with MetaCyc pathway annotations and merged across samples. Both unstratified and stratified pathway tables were created, and unmapped and unintegrated entries were excluded from these datasets. Filtering was applied based on abundance (retaining only pathways with a mean abundance of > 1 CPM across samples) and prevalence (retaining only pathways present in >5% of samples).

#### Statistical Analyses

All statistical analyses were performed using R^48^ (v4.3.2) via RStudio^49^. Principal component analyses were visualized using the ggplot2^50^ (v3.5.2) package. Linear mixed effect models were performed using lme4 package^51^ (v31.1.37) for model fitting and the lmerTest package^52^ (v3.1.3) for significance testing. Differential abundance analyses were performed using MaAsLin2^53^ (v1.16.0) with linear mixed models with patients included as a random effects. Heatmaps were generated using the pheatmap^54^ (v1.0.13) and ComplexHeatmap^55^ (v2.18.0) packages, with hierarchical clustering based on Euclidean distance and complete linkage. Repeated measures correlations (rmcorr) between microbiome and metabolome features were calculated using the rmcorr package^56^ (v0.7.0). False discovery rate correction for multiple testing was applied using the Benjamini–Hochberg method unless otherwise indicated, and only associations with an adjusted q < 0.05 were considered statistically significant. Differences in community composition were assessed using permutational multivariate analysis of variance (PERMANOVA) via adonis in the vegan package^57^ (v2.6.10), with 9,999 permutations and Bray-Curtis dissimilarities.

## Results

### FMTs significantly alter lipidomes of post-antibiotic rCDI patients.

Our analysis identified 397 non-bile acid lipids from 26 lipid classes across all patient samples (Table S6). To assess whether FMT significantly alters the fecal lipidome, we first conducted a comparison of overall lipid profiles from pre- and post-FMT samples. Principal component analysis (PCA) of log2-normalized lipid abundances revealed clear separations between pre-and post-FMT samples along major axis, which explained 26.6% and 11.6% of the total variance, respectively (Figure 1B). This compositional shift between pre- and post-FMT samples was statistically significant with patient as a stratifying factor, indicating a generalized restructuring of the lipidome following FMT (adonis, R^2^ = 0.1194, p = 0.001, permutations = 999). When the comparison was made across all sampled time points, the results were still significant and explained a larger proportion of the variance, suggesting that the lipidome not only undergoes substantial restructuring immediately post-FMT, but continues to undergo structural changes over time (R^2^ = 0.16209, p = 0.001, permutations = 999).

To identify temporal changes in specific lipid species, we utilized linear mixed-effect models (MEMs) across the time points. Of the 397 lipid species tested, 96 were significantly altered at one or more time points (FDR-adjusted p < 0.05 across time contrasts; Table S7). These included 24 lipids at 2 weeks, 56 lipids at 2 months, and 89 lipids at 6 months post-FMT. This model captured both short term and sustained lipidomic shifts, revealing dynamic lipidome remodeling over time. Of the significant lipids, 18 were sphingomyelins (SM), 15 ceramides (Cer), 12 acylcarnitines (AC), nine were fatty acyls (FA), seven were hexosylceramides (HexCer), seven were lysophosphatidylcholines (LPC), seven were phosphatidylglycerols (PG), five were phosphatidylethanolamines (PE), four were gangliosides (GMx), three were lysophosphatidylethanolamines (LPE), two were monogalactosyldiacylglycerols (MGDG), two were phosphatidylcholines (PC), two were 1-(1Z-alkenyl),2-acyl-glycerophosphocholine (PC-P), and one each were sulfoquinovosyldiacylglycerols (SQDG), fatty acyl esters of hydroxy fatty acid (FAHFA), and 1-(1Z-alkenyl),2-acyl-glycerophosphoethanolamine (PE-P). To further characterize the patterns of lipid changes over time, we performed hierarchical clustering on the subset of 96 MEM-significant lipids across all relevant samples. We observed two major lipid clusters (Figure 1C). Cluster one was comprised of 82 lipids that were elevated in pre-FMT samples and showed consistent decreases at 2 weeks that persisted through 6 months, while cluster two exhibited 14 lipids that were more variable or elevated post-FMT.

**Figure 2.**
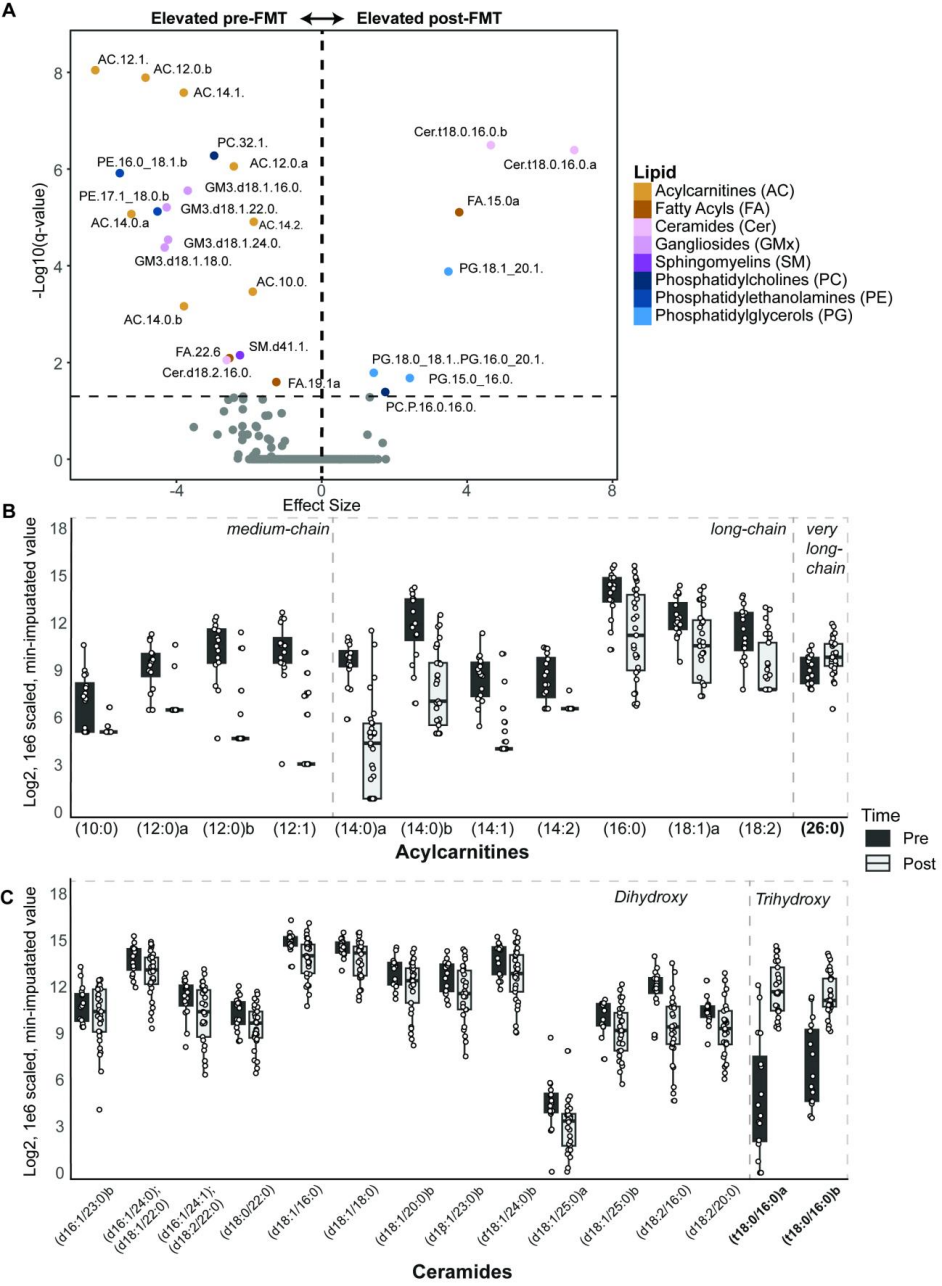
Medium-chain acylcarnitines and trihydroxy ceramides change the most post-FMT. (A) Volcano plot showing lipids that differed between pre- and post-FMT samples. (B) Boxplots showing changes over time in MEM significant acylcarnitines. (C) Boxplots showing changes over time in MEM significant ceramides. Statistical analysis was done on log_2_-transformed, 1 × 10^6^-scaled, minimum-imputed lipid data using a MEM model.

To complement the time-resolved mixed-effects modeling, which captured dynamic lipid trajectories, we next examined bulk pre- versus post-FMT differences using differential abundance analysis to identify the most prominent lipid changes associated with FMT. MaAsLin2 identified 25 lipids as significantly different, including seven enriched lipids post-FMT and 18 enriched lipids pre-FMT (Bonferroni-adjusted q < 0.05; Table S8). Effect sizes and directionality were visualized in a volcano plot (Figure 2A), which revealed that the majority of significant associations had negative coefficients, indicating depletions post-FMT. Top pre-FMT enriched lipids included eight medium and long acylcarnitine species, all of the assayed gangliosides, as well as an assortment of other lipid species. Post-FMT enriched lipids included both of the assayed trihydroxy ceramides, a fatty acyl, three PGs, and a PC.

### Medium and long-chain acylcarnitines and trihydroxy ceramides are the strongest drivers of lipidomic changes.

To determine the lipid classes most strongly associated with FMT induced changes, we analyzed the subset of lipids found to be most significant based on both the MEM and MaAsLin2 analyses. Among these, AC and Cer both demonstrated the most significant temporal shifts. Boxplots of all MEM-significant lipid species from these classes across timepoints reveal consistent patterns (Figure 2B–C). Specifically, medium and long-chain ACs ranging from ten to 18 carbons in chain length exhibited significant elevations in pre-FMT samples followed by a marked decrease at 2 weeks post-FMT, which was then sustained through 6 months. On the other hand, the longest chain ACs assayed, which were of 24 and 26 carbon chain lengths, both demonstrated marked increases post-FMT, although the 24 chain acylcarnitine was not significant. Overall, 12 of the total 15 ACs assayed showed significant temporal changes, suggesting that these metabolites are critical components of FMT associated metabolic shifts. Meanwhile, of the 24 Cers assayed, 15 changed significantly per the MEM-model. Of these, 13 Cers, all with dihydroxy bases, were elevated pre-FMT and declined post-FMT. However, two of the three trihydroxy Cers assayed (Cer(t18:0/16:0)a and Cer(t18:0/16:0)b) were substantially elevated post-FMT (Figure 2C).

To better understand the lipidomic remodeling occurring post-FMT, we examined the lipid subclasses most closely biochemically related to ACs and Cers. These included other FA and FAHFA species, as well as sphingolipids including GMxs, HexCers, and SMs. For the FAs, we observed that eight of 34 species exhibited declines post-FMT, while one species (FA(15:0)a) was elevated (Figure S1A). Only one of three assayed FAHFAs was MEM-significant (FAHFA(17:0/0–19:2)), and this species exhibited declines post-FMT (Figure S1B). Among sphingolipids, most species exhibited similar trends to dihydroxy Cers. Specifically, all four GMx species declined post-FMT (Figure S2A), seven of nine HexCer species declined post-FMT (Figure S2B), and 18 of 25 SM species declined post-FMT (Figure S2C).

### Enterobacteriaceae are strongly correlated with medium- and long-chain ACs, while four families are associated with very long-chain ACs.

**Figure 3.**
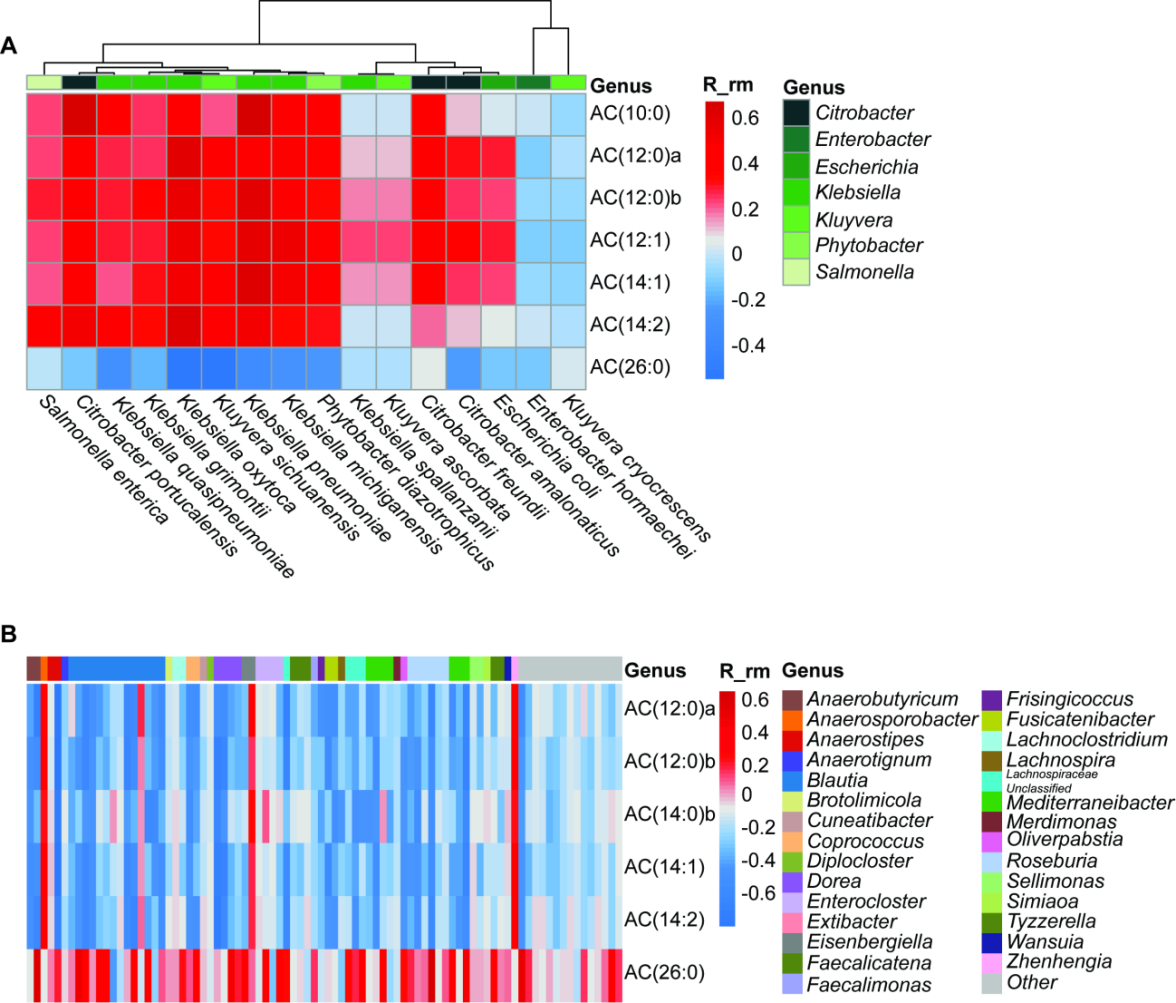
Enterobacteriaceae and Lachnospiraceae correlate inversely with very long-chain acylcarnitines (A) Medium- and long-chain acylcarnitines were positively correlated with Enterobacteriaceae species. (B) Dihydroxy ceramides were also positively correlated with Enterobacteriaceae. (C) Very long-chain acylcarnitines were correlated with Lachnospiraceae species. Correlations were calculated using repeated-measures correlation on MEM-significant lipids and bacterial species.

To investigate the association between microbial species and lipid species across the sampling points, we performed repeated measures correlation (rmcorr) analysis. We first examined all bacterial families (Figure S3), and this initial screen revealed that Enterobacteriaceae exhibited the strongest positive correlation with medium- and long-chain ACs (Figure S3). In contrast, four families (Coriobacteriaceae, an unclassified Eubacteriales family, Lachnospiraceae and Oscillospiraceae) showed the strongest inverse correlations with these same lipid species, and strong positive associations with the very long-chain acylcarnitine AC(26:0) (Figure S3). Based on previous literature implicating Lachnospiraceae in bile acid metabolism via BSHs and other lipid modifying enzymes, we selected this family as well as Enterobacteriaceae for further in-depth examination. Thus, we repeated this analysis at the species level for both Families (Figure 3A–B). Within Enterobacteriaceae, we observed that five out of six of the identified *Klebsiella* species as well as *Citrobacte*r, *Kluyvera, Salmonella*, and *Phytobacter* species were strongly associated with pre-FMT acylcarnitine lipids, while *Enterobacter hormaechei, Klebsiella Spallanzani, Kluyvera ascorbate* and *Kluyvera cryocrescens* had inverse trends. For those species which correlated with the medium- and long-chain ACs, all associations were constrained to ACs with a chain length of 14 carbons or fewer. Meanwhile, the 86 Lachnospiraceae species identified as significant were, for the most part, strongly associated with the very long-chain acylcarnitine AC26:0, although four species (*Anaerosporobacter mobilis, Blautia producta, Eisenbergiella tayi, and Zhenhengia yiuensis*) were associated with long-chain ACs of chain lengths 12 and 14 (Figure 3B).

### While Klebsiella and Escherichia dominate pre-FMT amino acid biosynthesis and utilization, Blautia and other Lachnospiraceae contribute to the majority of activity post-FMT.

**Figure 4.**
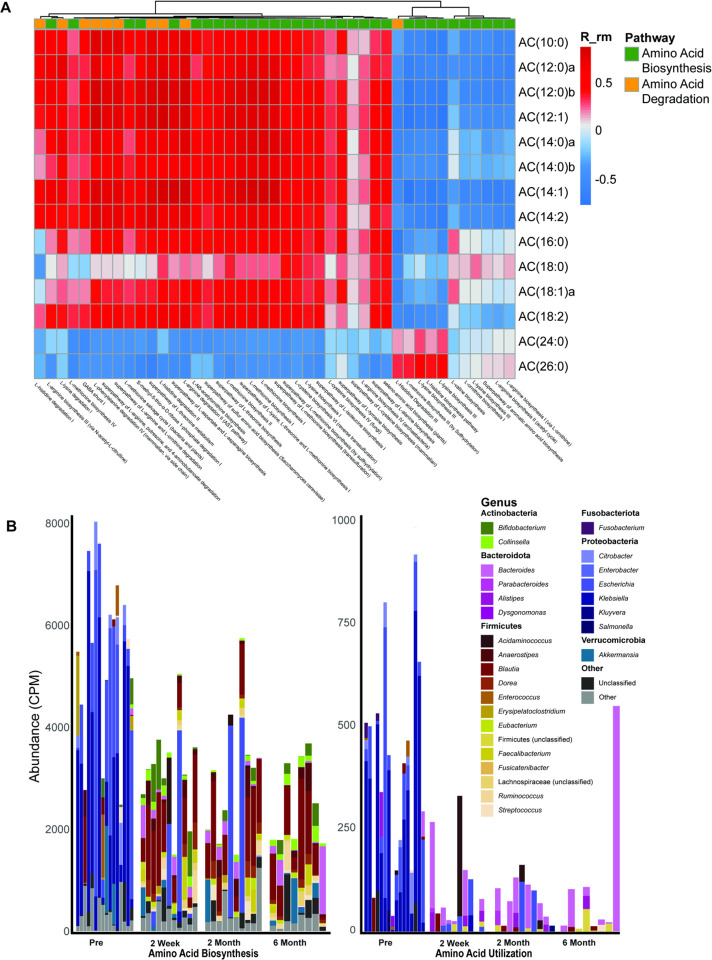
Amino acid pathways associate with Proteobacteria pre-FMT and with Firmicutes post-FMT. (A) Acylcarnitines of different chain lengths were correlated with significant amino acid biosynthesis and degradation pathways. (B) Stacked bar plots showing the bacterial genera encoding these biosynthesis and degradation pathways, with each column representing an individual patient. Analyses used repeated-measures correlation of MEM-significant lipids with amino acid pathways, and pathway assignments were split by genus.

To better understand host-microbe metabolic interactions, we assessed correlations between ACs and MEM significant microbial amino acid biosynthesis and utilization pathways (Figure 4A). We found that the medium- and long-chain pre-FMT associated ACs were significantly correlated with a much higher number of amino acid biosynthesis pathways and accounted for 32 out of the 43 significant amino acid utilization pathways (with the exception an L-histidine degradation pathway). Meanwhile, the five pathways most strongly correlated with very long-chain post-FMT associated ACs involved L-glutamine, L-histidine, L-lysine, and L-methionine biosynthesis. To identify the key microbial contributors to these associations, we analyzed stratified pathway abundances by genus and visualized them over time (Figure 4B). We observed that pre-FMT, amino acid biosynthesis and degradation pathways are driven by Enterobacteriaceae including *Escherichia* and *Klebsiella*. Post-FMT, amino acid pathways originate from a consortium of microbes, including Lachnospiraceae such as *Blautia*. We then performed a similar analysis for pathways associated with lipid biosynthesis and degradation and found that members of the *Escherichia* and *Klebsiella* genera contributed a substantial proportion of the total pathway abundance pre-FMT (80% ± 29%) (Figure S4). This contribution sharply decreased by two weeks post-FMT (31% ± 40%) and continued to decline at two months (20% ± 42%) and six months (4% ± 10%) post-FMT.

## Discussion

This study highlights the utility of extensive lipid profiling for 26 different lipid classes to investigate FMT outcomes by performing lipidomic analyses with LC-IMS-CID-MS^46^. This multidimensional approach integrates retention time, collision cross section, fragmentation information and accurate mass, enabling confident lipid annotation which we utilized to separate isomeric lipid species across multiple classes and observe fine structural differences. In this study, we were able to expand upon our previously untargeted longitudinal metabolomic analyses of 15 antibiotic-treated rCDI patients undergoing FMT procedures^31^ and verify the presence of global lipidomic remodeling following FMTs. Moreover, we were able to identify 96 lipid species (Table S7) with significant shifts, the majority of which have not been previously observed within the rCDI-FMT context. Of these lipids, the most dramatic shifts involved medium- and long-chain AC and sphingolipid species, most of which were elevated in post antibiotic, pre-FMT states and rapidly declined post-FMT. Meanwhile, trihydroxy Cers were most elevated in post-FMT samples. Using paired metagenomics, we identified that Enterobacteriaceae species were strongly associated with medium- and long-chain ACs, suggesting that these metabolites may be involved in active metabolic relationships that might increase susceptibility to *C. difficile* colonization.

Within the context of the literature, our observation that specific acylcarnitine species are the primary drivers of FMT-associated lipidomic differences align with and extend upon prior studies. ACs, which are fatty acids critical for cellular energy metabolism pathways such as β-oxidation, have long been associated with a plethora of gastrointestinal and inflammatory conditions including inflammatory bowel disease (IBD)^58–60^, diabetes^61,62^, cancer^63^, cardiovascular conditions^64^, and liver disease^65^. The mechanistic association between ACs and gastrointestinal inflammation has been directly supported by studies such as those of Lemons et al., which demonstrated that luminary gut increases in ACs are the result of increasing biliary secretions and intestinal damage associated release of host cellular materials^60^. They further demonstrated that ACs, specifically excluding very-long chain species, could directly promote the growth of Enterobacteriaceae species thought to play roles in IBD pathogenesis^60^. Within the context of CDI, several studies have linked elevated short- to long-chain ACs to CDI and rCDI susceptibility. For example, Li et al., prospectively examined juvenile primary CDI and assessed the differences between recurrers and non-recurrers, ultimately finding several acylcarnitine species to be elevated in the recurrers^66^. Corroborating these findings in humans, Dawkins et al., examined 53 patients with primary CDI and followed their outcomes post-antibiotic treatment^39^. By independently analyzing their published data, we also identified that the short chain ACs C6, C8, and C10 were substantially elevated in recurrers versus non-recurrers. Although these studies focus on primary CDI, they collectively suggests that ACs may be associated with colonization susceptibility. This is consistent with our observation of reduced ACs following successful FMT-treatments. Still, the association of these metabolites with Enterobacteriaceae requires further exploration. While our data is strongly suggestive of a possible cross-feeding mutualistic relationship as exhibited in Lemons et al., 2023 within the context of IBD^60^, it remains unproven that ACs directly exacerbate CDI outcomes or recurrence risk through these microbes. Additionally, it would benefit future researchers to expand the lipid library utilized in this study to include short and medium-chain ACs (e.g., with lengths of 2 to 12 carbons), as those have been identified by other researchers to perform important physiological functions but were not captured in our current analyses.

Another observation gleaned from this study were specific sphingolipids difference pre- vs. post-FMT. Sphingolipids, a class of lipids containing sphingoid bases and which are critical to signal transduction and cell membrane functions, have long been known to be both associated with host-microbial interactions^67^ as well as critical players in *C. difficile* pathogenesis^68^. Several pathogenic bacterial species can manipulate host sphingolipid-enzymes (e.g., acidic sphingomyelinase), driven cellular membranes remodeling such as elevations in Cers^69,70^, while *C. difficile* and other microbes can then use to facilitate attachment to cells. Indeed, several bacterial toxins including lipopolysaccharide (LPS) and possibly *C. difficile* toxin B can facilitate ceramide increases via a TLR4-dependent response that also sets of a pro-inflammatory signaling cascade^71–74^. These processes result in the accumulation of Cers, and it has been demonstrated through murine knockout studies that decreases or elevations of Cers of specific lengths (e.g., C16) can increase endotoxin lethality and exacerbate DSS colitis^75,76^. Beyond host pathways, bacterial-derived sphingolipids can reduce host sphingolipid production^77^, which could be hypothesized to alter host inflammatory signaling. For instance, in one IBD study, *Bacteroides* sphingolipids were decreased in the patients’ stool and negatively correlated with gut inflammation, with a trend of the opposite direction associated with host sphingolipids^36^. From these findings, we infer that the elevations in dihydroxy Cers, likely host-derived, observed pre-FMT are associated with active inflammatory processes and damage intestinal damage resulting in the release of host cellular materials. However, the drastic elevations in trihydroxy Cers post-FMT are more challenging to explain. As some bacterial species are known to synthesize various sphingolipids^77^ as well as dihydroceramides with a third hydroxyl-group^36^, this could be evidence of elevated bacterial contributions. However, the presence of trihydroxy Cers might also be suggestive of gut epithelial remodeling with an altered lipid composition. Further work incorporating healthy controls with variable levels of sphingolipid-producing commensal microbes may be one way to clarify the mechanisms leading to this phenomenon.

As lipids gain the increasing recognition as critical drivers of diverse biological and pathological processes, these endeavors are challenged by a lack of understanding of how specific lipids interact with both hosts and microbes physiologically as byproducts or direct contributors to altered health and disease states. Our improved lipidomic profiling approach had enabled us to form testable hypothesis about how FMT successfully restores colonization resistance in antibiotic-treated rCDI patients. Established direct mechanistic links, however, will require incorporating healthy controls to distinguish disease-specific metabolome and microbiome shift with respect to a baseline population.

To conclude, our lipidomic analyses not only confirmed global changes associated with FMT administration but also identified several lipidomic shifts that point to mechanistic hypotheses testable through *in vivo* and *in vitro* experimentation. In the future, directly testing these mechanisms through competition and colonization studies will be essential for designing the improved LBP therapeutic products that target the colonization susceptibility underlying rCDI.

## Supplementary Material

Supplementary Files

This is a list of supplementary files associated with this preprint. Click to download.

- SupplementalFigure1.tif
- SupplementalFigure2.tif
- SupplementalFigure3.tif
- SupplementalFigure4.tif
- SupplementalTablesFMTLipidsAbigailGancz8212025.xlsx

Supplemental Figures

**Figure S1:** Boxplots showing changes over time in (A) significant fatty acyls and (B) significant fatty acyl esters of hydroxyl fatty acids. Statistical analysis was done on log_2_-transformed, 1 × 10^6^-scaled, minimum-imputed lipid data using a MEM model.

**Figure S2:** Boxplots showing changes over time in (A) significant gangliosides, (B) significant hexosylceramides, and (C) significant sphingomyelins. Statistical analysis was done on log_2_-transformed, 1 × 10^6^-scaled, minimum-imputed lipid data using a MEM model.

**Figure S3:** Correlations between acylcarnitines and bacterial families. Analyses used repeated measures correlation of MEM-significant lipids and bacterial genera.

**Figure S4:** Stacked bar plots showing the bacterial genera encoding lipid associated biosynthesis and degradation pathways, with each column representing an individual patient.

## Figures and Tables

**Table 1:** Lipid Identification Modes and Number of Species

Lipid Classes	Abbreviation	Mode of Detection	# of Species
Acylcarnitines	CAR	Positive	15
Cholesteryl esters	CE	Positive	4
Ceramides	Cer	Negative	24
Diacylglycerols	DAG	Positive	9
Digalactosyldiacylglycerols	DGDG	Positive	11
Fatty Acyls	FA	Negative	34
Fatty Acyl Esters of Hydroxy Fatty Acid	FAHFA	Negative	3
Gangliosides	GMx	Negative	4
Hexosylceramides	HexCer	Negative	9
Lysophosphatidylcholines	LPC	Positive	27
Lysophosphatidylethanolamines	LPE	Negative	15
Lysophosphatidylglycerols	LPG	Negative	3
Lysophosphatidylinositols	LPI	Negative	4
Monogalactosyldiacylglycerols	MGDG	Positive	2
Phosphatidylcholines	PC	Positive	52
1-alkyl,2-acyl-glycerophosphocholine	PC O-	Positive	10
1-(1Z-alkenyl),2-acyl-glycerophosphocholine	PC P-	Positive	5
Phosphatidylethanolamines	PE	Negative	20
1-alkyl,2-acyl-glycerophosphoethanolamine	PE O-	Negative	2
1-(1Z-alkenyl),2-acyl-glycerophosphoethanolamine	PE P-	Negative	16
Phosphatidylglycerols	PG	Negative	30
Phosphatidylinositols	PI	Negative	16
Phosphatidylserines	PS	Negative	4
Sphingomyelins	SM	Positive	25
Sulfoquinovosyldiacylglycerols	SQDG	Positive	5
Triacylglycerols	TG	Positive	48

## Data Availability

Raw sequences from shallow shotgun sequencing have been deposited in the Sequence Read Archive (SRA) under BioProject ID PRJNA1055134. Data acquired from targeted metabolomics have been deposited in MassIVE under MSV00099241. Code required for statistical analysis are available in GitHub here: https://github.com/LooseGoose99/FMT_LipidPaper_2025. Other data and biological materials are available from the corresponding authors upon reasonable requests.
